# Diagnosis of an imported *Plasmodium ovale wallikeri* infection in Malaysia

**DOI:** 10.1186/s12936-015-1070-z

**Published:** 2016-01-06

**Authors:** Jonathan Wee Kent Liew, Rohela Mahmud, Lian Huat Tan, Yee Ling Lau

**Affiliations:** Department of Parasitology, Faculty of Medicine, University of Malaya, 50603 Kuala Lumpur, Malaysia; Sunway Medical Centre, Bandar Sunway, 46150 Petaling Jaya, Selangor Malaysia

**Keywords:** *Plasmodium ovale wallikeri*, PCR, Diagnosis, Malaysia, Southeast Asia

## Abstract

*Plasmodium ovale* is rare and not exactly known to be autochthonous in Malaysia. There are two distinct forms of the parasite, namely *P. ovale curtisi* (classic form) and *P. ovale wallikeri* (variant form). Here, the first sequence confirmed case of an imported *P. ovale wallikeri* infection in Malaysia is presented. Microscopy found *Plasmodium* parasites with morphology similar to *P. ovale* or *Plasmodium vivax* in the blood films. Further confirmation using polymerase chain reaction (PCR) targeting the small-subunit rRNA gene of the parasite was unsuccessful. Genus-specific PCR was then performed and the product was sequenced and analysed. Sequence analyses confirmed the aetiological agent as *P. ovale wallikeri*. New species-specific primers (rOVA1v and rOVA2v) were employed and *P. ovale wallikeri* was finally confirmed. The findings highlight the need to look out for imported malaria infections in Malaysia and the importance of a constantly updated and validated diagnostic technique.

## Background

Malaria is caused by the blood parasite *Plasmodium.Plasmodium falciparum*, *Plasmodium vivax*, *Plasmodium ovale curtisi, P. ovale wallikeri*, *Plasmodium knowlesi* and *Plasmodium malariae* are known to infect humans. Current molecular-based diagnostic methods enable sensitive and specific detection of the five human malaria parasites, even in subclinical cases. Improved diagnostic techniques using polymerase chain reaction (PCR) detected a spike in prevalence of overall malaria infections. The relatively insensitive microscopic diagnosis has underestimated the prevalence of this disease, including *P. ovale* infection, which was once believed to be comparatively rare. Review of a series of studies led to a probable conclusion that a 2 to >10 % prevalence of *P. ovale* might actually exist in the general population of Africa and Papua New Guinea and among malaria patients in Southeast Asia [1].

*Plasmodium ovale* infection, a tertian malaria, is difficult to diagnose microscopically, owing to the generally low parasite density in patients and the parasite’s morphology which resembles that of *P. vivax*. Additionally, it is a relapsing infection, which can occur long after the primary exposure to the parasite, initiated by latent parasites (hypnozoites) residing in the liver [2]. Moreover, dimorphism exists in the small-subunit rRNA (ssrRNA) gene of the parasite, causing some isolates to be undetectable by PCR but microscopically confirmed as *P. ovale* [3]. Further gene studies demonstrated two distinct, non-recombining sympatric species of the parasite [4]. Subsequently in 2010, Sutherland et al. [4] first coined *P. ovale curtisi* and *P. ovale wallikeri* as names for the classic and variant forms of *P.ovale*, respectively.

Hitherto, cases of *P. ovale wallikeri* infection have been described in certain countries in Southern Asia (e.g. Bangladesh and India) and almost throughout the whole of Southeast Asia [4–14]. In Malaysia currently, the most prevalent malaria parasite is *P. knowlesi* [15], with majority of the cases originating from Malaysian Borneo. Seven known cases of *P. ovale* were reported so far in Malaysia [15–18]. Here, the first sequence confirmed case of an imported *P. ovale wallikeri* infection in a patient in Malaysia is reported. Microscopy, nested PCR and sequence analyses of the ssrRNA gene confirmed the aetiological agent as *P. ovale wallikeri*.

## Case presentation

A 19-year-old, Nigerian college student presented to Sunway Medical Centre, Selangor, Malaysia with a 4-day history of fever associated with chills, rigors and body ache. Prior to onset of symptoms, he had gone back to Nigeria for 10 days during semester break and became unwell 5 days after returning to Malaysia. He was febrile upon admission with body temperature of 39.8 °C, pulse rate of 126 beats per minute and blood pressure of 128/87 mm Hg. There were no other significant physical findings noted. He recalled to have had three episodes of malaria during childhood, but was uncertain about the causative *Plasmodium* species. Blood investigation showed thrombocytopaenia with a platelet count of 71,000 per microlitre (normal: 150,000–400,000 per microlitre) and raised C-reactive protein (97 mg/L; normal: <5 mg/L). White blood cell count, haemoglobin and albumin levels were within the normal range. Dengue NS-1Ag, dengue IgM and IgG, *Leptospira* IgM, *Mycoplasma* antibody test and Widal-Weil-Felix test were all negative. Blood cultures did not yield any result.

**Fig. 1:**
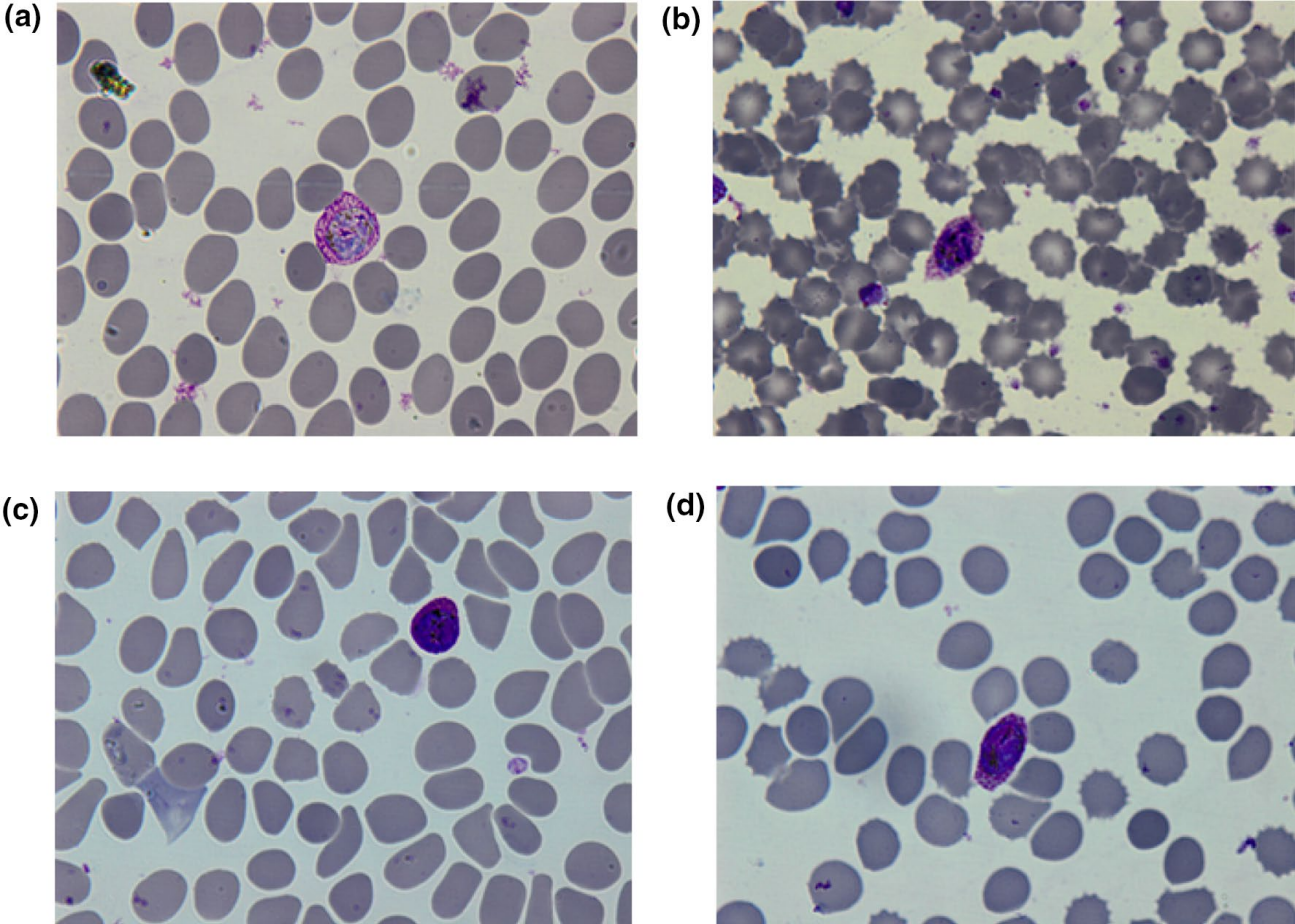
Microphotographs of giemsa-stained thin blood films of the patient viewed under 1000 ×  magnification. **a** Developing schizont. **b** A schizont with eight merozoites. **c**, **d** Gametocyte. Infected red blood cell is enlarged, oval in shape with presence of *Schuffner’s dots*, clearly seen in **b** and **d**

After three attempts using BinaxNOW^®^ Malaria Test (USA), a T2 Line appeared, while no T1 Line (specific for *P. falciparum* infection) was observed, suggesting vivax, malariae or ovale malaria or a mix of these. Microscopy on thick and thin blood films detected the presence of malaria parasites suggesting *P. ovale* or *P. vivax* (Fig. 1), with 0.27 % parasitaemia.

**Fig. 2:**
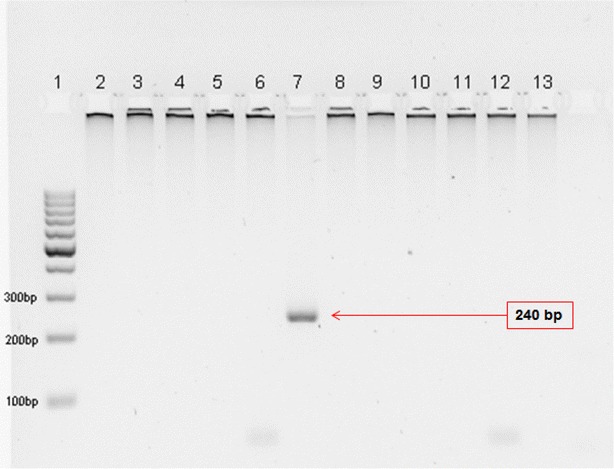
2 % agarose gel electrophoresis of Nest 2 PCR showing an amplified product with the genus-specific primers. *1* 100 bp DNA ladder; *2* rFAL1/rFAL2 (*P. falciparum*); *3* rOVA1/rOVA2 (*P. ovale*); *4* rMAL1/rMAL2 (*P. malariae*); *5* rVIV1/rVIV2 (*P. vivax*); *6* Pmk8/Pmkr9 (*P. knowlesi*); *7* rPLU3/rPLU4 (*Plasmodium* spp.);* 8–13* no template control of *P. falciparum*, *P. ovale*, *P. malariae*, *P. vivax*, *P. knowlesi*, and *Plasmodium* spp., respectively

DNA was extracted from whole blood collected in EDTA tube using the DNeasy^®^ Blood and Tissue Kit (Qiagen, Germany). Four microlitres of the purified DNA was used in the primary PCR reaction, followed by a secondary PCR amplification using standard nested PCR method [16, 19] (Table 1). The PCR result was negative for all reactions except the reaction using genus-specific primers, rPLU3 and rPLU4. Gel electrophoresis showed a band of 240 bp, corresponding to the product of the genus-specific nest 2 PCR (Fig. 2). At the same time, NM-PCR, a nested multiplex malaria PCR and NG-PCR, a nested PCR for *Plasmodium* genus amplification were performed (Table 1), as previously described [20]. NG-PCR also successfully amplified a positive product. The positive products of both PCRs were gel purified using QIAquick^®^ Gel Extraction Kit (Qiagen, Germany) and cloned into pGEM^®^-T vector (Promega, USA). For each PCR product, ten clones were screened and three positive clones were randomly selected for plasmid sequencing. Plasmids were extracted using QIAprep^®^ Spin Miniprep Kit (Qiagen, Germany) and sequenced using BigDye^®^ Terminator v3.1 in ABI PRISM^®^ 3730*xl* DNA Analyzer (Applied Biosystems). No definitive result was obtained from NM-PCR due to non-specific bands observed on the gel.

**Table 1:** Description of PCR protocols used in this report

Reaction	Primer	Annealing temperature (°C)	Product size (bp)	Target species	Reference
*Nested PCR*					
Nest 1^a^	rPLU1/rPLU5	55	≈1670	*Plasmodium* spp.	[19]
Nest 2^b^	rPLU3/rPLU4		240	*Plasmodium* spp.	
	rFal1/rFal2		205	*P. falciparum*	
	rVIV1/rVIV2		120	*P. vivax*	
	rMAL1/rMAL2		145	*P. malariae*	
	rOVA1/rOVA2	58	787–789	*P. ovale curtisi*	
	Pmk8/Pmkr9		153	*P. knowlesi*	[16]
	rOVA1v/rOVA2v		782	*P. ovale wallikeri*	[3]
	rOVA1WC/rOVA2WC		659–662	*P. ovale curtisi* and *P. ovale wallikeri*	[26]
*Mixed P. ovale* ^c^	rOVA1/rOVA2 and rOVA1v/rOVA2v	58	782–789	*P. ovale curtisi* and *P. ovale wallikeri*	[3]
*NM*- *and NG*-*PCR*					
Primary PCR^d^	UNR/PLF	58	783–821	Universal	[20]
NM PCR^e^	New PLFshort with	53		*Plasmodium* spp.	
	MARshort		241	*P. malariae*	
	FARshort		370	*P. falciparum*	
	OVRshort		407	*P. ovale*	
	VIRshort		476	*P. vivax*	
NG-PCR^f^	NewPLFshort/NewRevshort	53	735–773	*Plasmodium* spp.	

^a^Cycling condition: Initial denaturation, 94 °C for 4 min; 35 cycles of 94 °C for 1 min, 55 °C for 1 min and 72 °C for 1 min; final extension 72 °C for 10 min. Four µL of DNA is used in a 25 µL reaction volume containing 4 mM magnesium chloride, 0.2 mM of each dNTPs and 1 Unit of Taq polymerase

^b^Cycling condition: Initial denaturation, 94 °C for 4 min; 35 cycles of 94 °C for 1 min, 58 °C for 1 min and 72 °C for 1 min; final extension 72 °C for 10 min. Four µL of nest one product is used in a 25 µL reaction volume containing 4 mM magnesium chloride, 0.2 mM of each dNTPs and 1 Unit of Taq polymerase

^c^Cycling condition is the same as Nest 2 PCR^b^. Primer sets rOVA1/rOVA2 and rOVA1v/rOVA2v are mixed in equimolar in a single PCR reaction tube. Four µL of nest one product is used in a 25 µL reaction volume containing 4 mM magnesium chloride, 0.2 mM of each dNTPs and 1 Unit of Taq polymerase

^d^Cycling condition: Initial denaturation, 94 °C for 5 min; 35 cycles of 94 °C for 1 min, 58 °C for 1 min and 72 °C for 1 min; final extension 72 °C for 10 min. Five µL of DNA is used in a 50 µL reaction volume containing 4 mM magnesium chloride, 0.2 mM of each dNTPs and 2 Units of Taq polymerase

^e^Cycling condition: Initial denaturation, 94 °C for 5 min; 35 cycles of 94 °C for 1 min, 53 °C for 1 min and 72 °C for 1 min; final extension 72 °C for 10 min. Two µL of Primary PCR product is used in a 25 µL reaction volume containing 4 mM magnesium chloride, 0.2 mM of each dNTPs and 1 Unit of Taq polymerase

^f^Cycling condition is the same as NM-PCR^e^. Two µL of Primary PCR product is used in a 50 µL reaction volume containing 4 mM magnesium chloride, 0.2 mM of each dNTPs and 2 Units of Taq polymerase

**Fig. 3:**
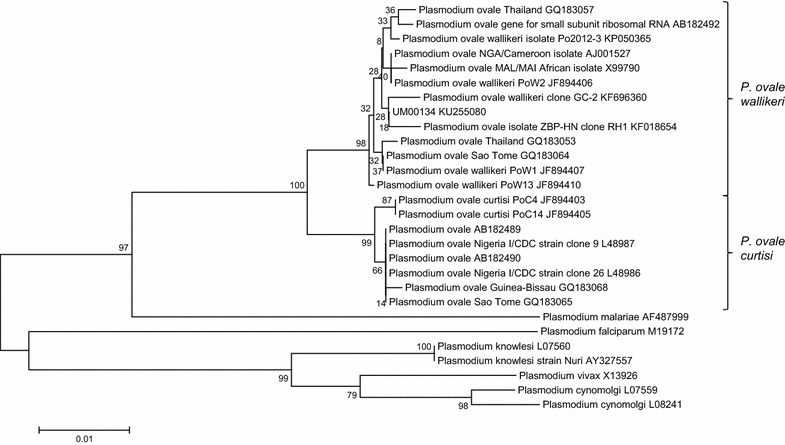
Phylogenetic analysis of the NG-PCR product (UM00134). The phylogenetic tree is constructed using the Neighbor-Joining methods and Jukes–Cantor model, with Bootstrap value of 1000. The patient isolate, UM00134 is clustered with *P. ovale wallikeri* isolates. GenBank accession number is given after each isolate’s name

Sequencing data between clones were similar except for a few nucleotide differences. A consensus sequence was generated from the plasmid sequences of the clones for each PCR product and analysed using NCBI BLAST [21]. The result showed a 99 % identity to the ssrRNA gene of *P. ovale wallikeri* (GenBank accession number: AB182492.1, KF696360.1, KF018654.1). Subsequently, the full nucleotide sequence (746 bp) of the product from NG-PCR (herein named UM00134; GenBank accession number: KU255080) was compared to those found in the GenBank in a phylogenetic analysis using the MEGA 6.06 program [22]. Sequences were trimmed and aligned using Clustal W [23]. A phylogenetic tree was constructed using the neighbour-joining methods [24] and Jukes-Cantor model [25], with a Bootstrap value of 1000 (Fig. 3). Sequence analyses confirmed that the infecting *Plasmodium* species was *P. ovale wallikeri*.

**Fig. 4:**
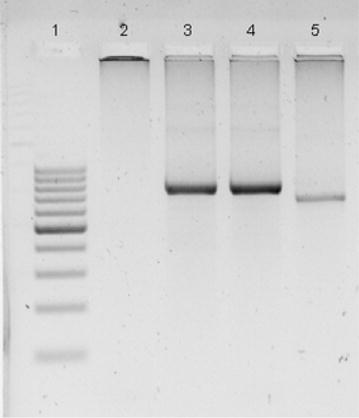
Detection of *P. ovale wallikeri* using primer sets rOVA1/rOVA2, rOVA1v/rOVA2v, mixture of both primer sets and rOVA1WC/rOVA2WC. A 2 % agarose gel electrophoresis was performed. Parasite DNA purified from patient blood is used as template of PCR. *1* 100 bp DNA ladder; *2* rOVA1/rOVA2 (*P. ovale curtisi*); *3* rOVA1v/rOVA2v (*P. ovale wallikeri*); *4* Mixture of primer sets rOVA1/rOVA2 and rOVA1v/rOVA2v (both forms of *P. ovale*); *5* rOVA1WC/rOVA2WC (both forms of *P. ovale*)

In light of this finding, primers rOVA1v and rOVA2v which are specific for *P. ovale wallikeri* [3], were used in the Nest 2 PCR reaction under the same condition as mentioned in Table 1. Primers rOVA1 and rOVA2 were included to exclude *P. ovale curtisi* infection. Parallel to that, a mixture of primer sets rOVA1/rOVA2 and rOVA1v/rOVA2v, besides primer set rOVA1WC/rOVA2WC [26] which are able to detect both *P. ovale* forms were also employed. Positive products were obtained from the PCR. The mixture of these primer sets in a single Nest 2 PCR reaction and primer set rOVA1WC/rOVA2WC were able to detect *P. ovale wallikeri* (Fig. 4).

The patient was immediately treated with a course of Riamet^®^ (artemether/lumefantrine) following detection of malaria parasites. His symptoms improved within 24 h of treatment but blood film for malaria became negative only after 36 h of treatment. However his platelet count seems to be slow to recover while C-reactive protein, which is known as a marker for severity in malaria [27], improved steadily following treatment. Primaquine 30 mg base daily was added 2 days after Riamet. He requested for discharge and agreed to complete the two-week course of primaquine at home. He continued to improve clinically post-discharge but could not afford to repeat his blood tests and dropped out from subsequent out-patient follow up visit.

## Consent

The study was approved by the University of Malaya Medical Centre Medical Ethics Committee (MEC Ref. No: 817.18).

## Discussion

In Malaysia, two of the seven ovale malaria were reported to be caused by *P. ovale curtisi* [18]. No known ovale infection in the country was sequence-confirmed to be caused by *P. ovale wallikeri*, up till now. Diagnosis of ovale malaria is a challenge, due to the parasite’s low density and close morphological resemblance to *P. vivax*. Both the classic and variant forms of *P. ovale* are also morphologically indistinguishable from each other [26]. Rapid diagnostic tests (RDT) have aided in the rapid diagnosis of malaria and other infectious diseases. Nonetheless, their apparent ineffectiveness in detecting *P. ovale* turns out to be well documented [28, 29]. In this case, the result of the RDT, BinaxNOW^®^ Malaria Test was negative twice before a faint line indicating *Plasmodium* species infection appeared on the third attempt.

Nested PCR for molecular detection of *Plasmodium* spp. [30] has emerged as a standard molecular method for malaria diagnosis. However, the primers for *P. ovale* detection (rOVA1/rOVA2) were unable to identify the variant form of the parasite, until a new set of primers (rOVA1v/rOVA2v) specific for *P. ovale wallikeri* and a set of primers (rOVA1WC/rOVA2WC) to detect both forms were introduced [3, 26]. The initial negative PCR result using the species-specific primers and subsequent sequencing analyses were indicative of *P. ovale wallikeri* infection, prompting the use of these new primers. Besides, primer sets rOVA1/rOVA2 and rOVA1v/rOVA2v were mixed to detect both *P. ovale wallikeri* and *P. ovale curtisi* in a single Nest 2 duplex PCR reaction. The mixed primers and primer set rOVA1WC/rOVA2WC in the PCR reaction appeared to be a mean for quick and cost-efficient detection of both species of *P. ovale* [26], and therefore should replace primers rOVA1/rOVA2 for routine diagnosis of malaria.

The accurate diagnosis of *P. ovale* infection, in fact of all malaria infection, is crucial to avoid the delay of treatment and clinical complications. Though ovale malaria is thought to be rather benign, associated severe complications such as acute respiratory distress syndrome (ARDS) [31, 32], splenic complication [33], acute renal failure and ultimately, death [18, 34] have been reported. There are certain clinical features slightly distinctive of the two different forms of *P. ovale*. The latency periods of *P. ovale curtisi* and *P. ovale wallikeri* infection are reported to be 66.1–111.1 days and 28.9–57 days, respectively, with one case of *P. ovale curtisi* infection displaying an extraordinarily long latency period of 1083 days [35]. This shorter latency period may lead to higher relapse rate of *P. ovale wallikeri* infection. A trend toward a shorter stay in Africa and shorter period of time between onset of symptoms and diagnosis was found among patients with *P. ovale wallikeri* infection, possibly reflecting easier transmission, shorter latency or higher relapse rates [6]. In addition, patients with *P. ovale wallikeri* infection have significantly more severe thrombocytopaenia and relatively higher parasitaemia than those with *P. ovale curtisi* infection. Other non-significant, differentiating features include shorter pre-patent period, lower albumin level, higher body temperature and more markers of haemolysis in *P. ovale wallikeri* infection compared to that of *P. ovale curtisi* [6]. Taken together, it may seem that management of *P. ovale wallikeri* could be slightly more complicated than *P. ovale curtisi*.

*Plasmodium ovale* is not exactly known to be autochthonous in Malaysia. It is absolutely undesirable for *P. ovale* to be transmitting within the country, especially when Malaysia is progressing towards elimination of malaria by 2020. Relapsing ovale infection is often asymptomatic, detected only by continued examination of blood films [2]. In other words, human reservoir of *P. ovale* may stay under the radar. This poses a threat to the public health because natural transmission of ovale malaria in Malaysia is possible in view of the availability of two vectors, *Anopheles maculatus* [36] and *Anopheles dirus* [37] which have been shown to be good experimental vectors for *P. ovale*. Nolder et al. [35] also found that, compared to other human malaria, ovale malaria is less likely to be prevented by chemoprophylaxis. Furthermore, as pointed out by Mellon et al. [38], with non-specific clinical signs as well as long latency period of *P. ovale* infection [2, 6, 35], what then should the maximum exclusion period be for blood donors who had travelled to endemic areas? Transfusion-transmitted *P. ovale* infection had been reported and can be severe [39].

It was not discernable if the patient was experiencing a relapse or acute malaria. Prepatent period (time between sporozoite inoculation and the first detection of parasites in blood) for ovale malaria is reported to be 12–20 days [2]. Lastly, a similar case was reported by Li et al. [29], whereby *P. ovale wallikeri* infection was diagnosed in a Chinese worker returning from West Africa. This and other increasing worldwide reports of *P. ovale* infections [40–43] and misdiagnosed cases [7] urge the health and research sectors to devise and utilise accurate techniques to diagnose this infectious disease.

## Conclusions

The current findings highlight the need for continuously updating and validating diagnostic techniques for malaria. Complete history taking is of utmost importance in patients as it aids diagnosis, especially in the case of *P. ovale* infection where it can remain latent and asymptomatic for a long period of time. Imported cases of malaria are a concern for public health, especially in countries where malaria is non-existent or has been eliminated. With Malaysia’s open policy and the ease of travel, healthcare personnel should be made aware of the possible imported infections entering the country.

## References

[1] Mueller I, Zimmerman PA, Reeder JC (2007). *Plasmodium malariae* and *Plasmodium ovale*–the “bashful” malaria parasites. Trends Parasitol.

[2] Collins WE, Jeffery GM (2005). *Plasmodium ovale*: parasite and disease. Clin Microbiol Rev.

[3] Calderaro A, Piccolo G, Perandin F, Gorrini C, Peruzzi S, Zuelli C (2007). Genetic polymorphisms influence *Plasmodium ovale* PCR detection accuracy. J Clin Microbiol.

[4] Sutherland CJ, Tanomsing N, Nolder D, Oguike M, Jennison C, Pukrittayakamee S (2010). Two nonrecombining sympatric forms of the human malaria parasite *Plasmodium ovale* occur globally. J Infect Dis.

[5] Fuehrer HP, Habler VE, Fally MA, Harl J, Starzengruber P, Swoboda P (2012). *Plasmodium ovale* in Bangladesh: genetic diversity and the first known evidence of the sympatric distribution of *Plasmodium ovale**curtisi* and *Plasmodium ovale**wallikeri* in southern Asia. Int J Parasitol.

[6] Rojo-Marcos G, Rubio-Muñoz Jé M, Ramírez-Olivencia G, García-Bujalance S, Elcuaz-Romano R, Díaz-Menéndez M (2014). Comparison of imported *Plasmodium ovale**curtisi* and *P. ovale**wallikeri* infections among patients in Spain, 2005–2011. Emerg Infect Dis.

[7] Chavatte J-M, Tan S, Snounou G, Lin R (2015). Molecular characterization of misidentified *Plasmodium ovale* imported cases in Singapore. Malar J.

[8] Incardona S, Chy S, Chiv L, Nhem S, Sem R, Hewitt S (2005). Large sequence heterogeneity of the small subunit ribosomal RNA gene of *Plasmodium ovale* in Cambodia. Am J Trop Med Hyg.

[9] Toma H, Kobayashi J, Vannachone B, Arakawa T, Sato Y, Nambanya S (1999). *Plasmodium ovale* infections detected by PCR assay in Lao PDR. Southeast Asian J Trop Med Public Health.

[10] Wickremasinghe R, Galapaththy GN, Fernando WA, de Monbrison F, Wijesinghe RS, Mendis KN (2008). An indigenous case of *Plasmodium ovale* infection in Sri Lanka. Am J Trop Med Hyg.

[11] Win TT, Lin K, Mizuno S, Zhou M, Liu Q, Ferreira MU (2002). Wide distribution of *Plasmodium ovale* in Myanmar. Trop Med Int Health.

[12] Zhou M, Liu Q, Wongsrichanalai C, Suwonkerd W, Panart K, Prajakwong S (1998). High prevalence of *Plasmodium malariae* and *Plasmodium ovale* in malaria patients along the Thai-Myanmar border, as revealed by acridine orange staining and PCR-based diagnoses. Trop Med Int Health.

[13] Baird JK (1990). Purnomo, Masbar S. *Plasmodium ovale* in Indonesia. Southeast Asian J Trop Med Public Health.

[14] Cabrera BD, Arambulo PV (1977). Malaria in the Republic of the Philippines: a review. Acta Trop.

[15] Yusof R, Lau YL, Mahmud R, Fong MY, Jelip J, Ngian HU (2014). High proportion of knowlesi malaria in recent malaria cases in Malaysia. Malar J.

[16] Singh B, Lee KS, Matusop A, Radhakrishnan A, Shamsul SSG, Cox-Singh J (2004). A large focus of naturally acquired *Plasmodium knowlesi* infections in human beings. Lancet.

[17] Lim YAL, Mahmud R, Chew CH, T T, Chua KH (2010). *Plasmodium ovale* infection in Malaysia: first imported case. Malar J.

[18] Lau YL, Lee WC, Tan LH, Kamarulzaman A, Syed Omar SF, Fong MY (2013). Acute respiratory distress syndrome and acute renal failure from *Plasmodium ovale* infection with fatal outcome. Malar J.

[19] Singh B, Bobogare A, Cox-Singh J, Snounou G, Abdullah MS, Rahman HA (1999). A genus- and species-specific nested polymerase chain reaction malaria detection assay for epidemiologic studies. Am J Trop Med Hyg.

[20] Ta TH, Hisam S, Lanza M, Jiram AI, Ismail N, Rubio JM (2014). First case of a naturally acquired human infection with *Plasmodium* cynomolgi. Malar J.

[21] Altschul SF, Gish W, Miller W, Myers EW, Lipman DJ (1990). Basic local alignment search tool. J Mol Biol.

[22] Tamura K, Stecher G, Peterson D, Filipski A, Kumar S (2013). MEGA6: molecular evolutionary genetics analysis version 6.0. Mol Biol Evol.

[23] Thompson JD, Higgins DG, Gibson TJ, Clustal W (1994). Improving the sensitivity of progressive multiple sequence alignment through sequence weighting, position-specific gap penalties and weight matrix choice. Nucleic Acids Res.

[24] Saitou N, Nei M (1987). The neighbor-joining method: a new method for reconstructing phylogenetic trees. Mol Biol Evol.

[25] Jukes TH, Cantor CR, Munro HN (1969). Evolution of protein molecules. Mammalian protein metabolism III.

[26] Fuehrer HP, Stadler MT, Buczolich K, Bloeschl I, Noedl H (2012). Two techniques for simultaneous identification of *Plasmodium ovale**curtisi* and *Plasmodium ovale**wallikeri* by use of the small-subunit rRNA gene. J Clin Microbiol.

[27] Paul R, Sinha PK, Bhattacharya R, Banerjee AK, Raychaudhuri P, Mondal J (2012). Study of C reactive protein as a prognostic marker in malaria from Eastern India. Adv Biomed Res.

[28] Bauffe F, Desplans J, Fraisier C, Parzy D (2012). Real-time PCR assay for discrimination of *Plasmodium ovale**curtisi* and *Plasmodium ovale**wallikeri* in the Ivory Coast and in the Comoros Islands. Malar J.

[29] Li Y, Wang G, Sun D, Meng F, Lin S, Hu X (2013). A case of *Plasmodium ovale**wallikeri* infection in a Chinese worker returning from West Africa. Korean J Parasitol.

[30] Snounou G, Viriyakosol S, Zhu XP, Jarra W, Pinheiro L, do Rosario VE (1993). High sensitivity of detection of human malaria parasites by the use of nested polymerase chain reaction. Mol Biochem Parasitol.

[31] Lee EY, Maguire JH (1999). Acute pulmonary edema complicating ovale malaria. Clin Infect Dis.

[32] Rojo-Marcos G, Cuadros-Gonzalez J, Mesa-Latorre JM, Culebras-Lopez AM, de Pablo-Sanchez R (2008). Acute respiratory distress syndrome in a case of *Plasmodium ovale* malaria. Am J Trop Med Hyg.

[33] Cinquetti G, Banal F, Rondel C, Plancade D, de Saint Roman C, Adriamanantena D (2010). Splenic infarction during *Plasmodium ovale* acute malaria: first case reported. Malar J.

[34] Hachimi MA, Hatim EA, Moudden MK, Elkartouti A, Errami M, Louzi L (2013). The acute respiratory distress syndrome in malaria: is it always the prerogative of *Plasmodium* falciparum?. Rev Pneumol Clin.

[35] Nolder D, Oguike MC, Maxwell-Scott H, Niyazi HA, Smith V, Chiodini PL, et al. An observational study of malaria in British travellers: *Plasmodium ovale**wallikeri* and *Plasmodium ovale**curtisi* differ significantly in the duration of latency. *BMJ Open.* 2013;3:5. doi:10.1136/bmjopen-2013-002711. 10.1136/bmjopen-2013-002711 PMC3657643 23793668

[36] Chin W, Contacos PG, Buxbaum JN (1966). The transmission of a West African strain of *Plasmodium ovale* by *Anopheles freeborni* and *Anopheles maculatus*. Am J Trop Med Hyg.

[37] Coatney GR, Collins WE, Warren M, Contacos PG (1971). The primate malarias.

[38] Mellon G, Ficko C, Thellier M, Kendjo E, Aoun O, Adriamanantena D (2014). Two cases of late *Plasmodium ovale* presentation in military personnel. J Travel Med.

[39] Haydoura S, Mazboudi O, Charafeddine K, Bouakl I, Baban TA, Taher AT (2011). Transfusion-related *Plasmodium ovale* malaria complicated by acute respiratory distress syndrome (ARDS) in a non-endemic country. Parasitol Int.

[40] de Laval F, Simon F, Bogreau H, Rapp C, Wurtz N, Oliver M (2014). Emergence of *Plasmodium ovale* malaria among the French armed forces in the Republic of Ivory Coast: 20 years of clinical and biological experience. Clin Infect Dis.

[41] Li M, Xia Z, Yan H (2014). New type of SSUrDNA sequence was detected from both *Plasmodium ovale**curtisi* and *Plasmodium ovale**wallikeri* samples. Malar J.

[42] Castellanos Mí E, Díaz S, Parsons E, Peruski LF, Enríquez F, Ramírez JL (2015). First imported *Plasmodium ovale* malaria in Central America: case report of a Guatemalan soldier and a call to improve its accurate diagnosis. Mil Med Res.

[43] Chaturvedi N, Bhandari S, Bharti PK, Basak SK, Singh MP, Singh N (2015). Sympatric distribution of *Plasmodium ovale**curtisi* and *P. ovale**wallikeri* in India: implication for the diagnosis of malaria and its control. Trans R Soc Trop Med Hyg.

